# Immunoregulatory and neuroprotective activity of ovocystatin

**DOI:** 10.1192/j.eurpsy.2022.1802

**Published:** 2022-09-01

**Authors:** B. Stańczykiewicz, J. Gburek, K. Gołąb, B. Konopska, A. Zabłocka

**Affiliations:** 1 Wroclaw Medical University, Department Of Psychiatry, Wroclaw, Poland; 2 Wroclaw Medical University, Department Of Pharmaceutical Biochemistry, Wroclaw, Poland; 3 Hirszfeld Institute of Immunology and Experimental Therapy, Polish Academy of Sciences, Department Of Microbiology, Wroclaw, Poland

**Keywords:** ovocystatin, Immunoactivation, Neurogenesis

## Abstract

**Introduction:**

Ovocystatin has beneficial properties for cognitive function in young rats and might prevents aging-related cognitive impairment in older animals, as well as reduces memory decline in APP/PS1 mice model.

**Objectives:**

Our study aimed at assessing the impact of ovocystatin on microglia activation and neurogenesis.

**Methods:**

Immunoactivation: Mouse wild type microglia were stimulated with ovocystatin at dose of 100 micrograms/ml. The effect of ovocystatin on nitric oxide production and interleukin 1 beta secretion were determined. Neurogenesis: Primary rat hippocampal neurons of H19-7 cell line was used. The impact of ovocystatin on proliferation, nitric oxide production, and expression of markers of neurogenesis: microtubule-associated protein 2 (MAP2, isoforms A/B and C/D) and Synapsin 1, were determined.

**Results:**

It was shown that ovocystatin does not stimulate microglial cells to produce inflammatory mediators. Whereas, no toxic effect of ovocystatin (1-100 ug/ml) on H19-7 cells viability, and dose-dependent down-regulation of proliferation were demonstrated. It was also shown that in primary hippocampal neurons of H19-7 cells incubated with ovocystatin (100 micrograms/ml), the expression level of MAP2 C/D (75kDa) - characteristic form of immature neurons is unchanged. However, the increased expression of MAP2 A/B protein (280 kDa) – characteristic for mature neurons was observed after 6 and 24h incubation with ovocystatin. Relatively to MAP2 A/B, increased expression of synapsin 1 was observed.

**Conclusions:**

The ovocystatin might be a potential activator of molecular mechanisms in primary hippocampal neurons, participating in regulation of neurogenesis. Nevertheless, further studies are needed.

**Disclosure:**

No significant relationships.

